# Deposition of Visible Light-Active C-Doped Titania Films via Magnetron Sputtering Using CO_2_ as a Source of Carbon

**DOI:** 10.3390/nano7050113

**Published:** 2017-05-16

**Authors:** Rachan Klaysri, Marina Ratova, Piyasan Praserthdam, Peter J. Kelly

**Affiliations:** 1 Center of Excellence on Catalysis and Catalytic Reaction Engineering, Department of Chemical Engineering, Faculty of Engineering, Chulalongkorn University, Bangkok 10330, Thailand; Shun_RK@hotmail.co.th (R.K.); Piyasan.P@chula.ac.th (P.P.); 2 Surface Engineering Group, School of Engineering, Manchester Metropolitan University, Manchester M1 5GD, UK; peter.kelly@mmu.ac.uk

**Keywords:** carbon doping, titanium dioxide, carbon dioxide, magnetron sputtering, photocatalysis, visible light, methylene blue, stearic acid

## Abstract

Doping of titanium dioxide with p-block elements is typically described as an efficient pathway for the enhancement of photocatalytic activity. However, the properties of the doped titania films depend greatly on the production method, source of doping, type of substrate, etc. The present work describes the use of pulsed direct current (pDC) magnetron sputtering for the deposition of carbon-doped titania coatings, using CO_2_ as the source of carbon; ratios of O_2_/CO_2_ were varied through variations of CO_2_ flow rates and oxygen flow control setpoints. Additionally, undoped Titanium dioxide (TiO_2_) coatings were prepared under identical deposition conditions for comparison purposes. Coatings were post-deposition annealed at 873 K and analysed with scanning electron microscopy (SEM), X-ray diffreaction (XRD), atomic force microscopy (AFM), and X-ray photoelectron spectroscopy (XPS). The photocatalytic properties of the thin films were evaluated under ultraviolet (UV) and visible light irradiation using methylene blue and stearic acid decomposition tests. Photoinduced hydrophilicity was assessed through measurements of the water contact angle under UV and visible light irradiation. It was found that, though C-doping resulted in improved dye degradation compared to undoped TiO_2_, the UV-induced photoactivity of Carbon-doped (C-doped) photocatalysts was lower for both model pollutants used.

## 1. Introduction

Titanium dioxide (TiO_2_) photocatalysts have attracted a lot of attention since the discovery of their properties through photocatalytic water splitting reactions in 1972 [1]. Over the past few decades titanium dioxide-based photocatalyts have been the subject of extensive research efforts, due to their low cost, low toxicity, and high chemical- and photo-stability. Anatase is the crystalline phase of titania that is typically referred to as being the most photocatalytically active, as compared to the other phases of TiO_2_; rutile and brookite. Despite a variety of applications of photocatalytic materials being proposed (e.g., building materials, antimicrobial surfaces, self-cleaning surfaces, waste water treatment, etc.), the practical application of photocatalytic materials still remains quite limited [2,3]. This is due to several drawbacks of titania, such as low photonic efficiency and the relatively high band gap value. Also, the short lifetime and fast recombination rates of the photogenerated charge carriers make TiO_2_ photocatalysts generally unsuitable for high throughput applications, e.g., the purification of heavily polluted industrial wastewater. The band gap value of 3.2 eV (for anatase) means that only UV light with a wavelength of 387 nm or below can be used for the photoactivation of titania. As UV constitutes just under 4% of the solar spectrum, for many practical applications it is necessary to shift the photocatalytic activity of titanium dioxide into the visible range.

Extensive work in this direction started with the pioneering work of Asahi and co-workers [4] who showed that the visible light response of titanium dioxide can be significantly enhanced through nitrogen doping. Consequently, the doping of TiO_2_ with p-block elements still remains a popular method for the synthesis of visible light photocatalysts, along with other techniques, such as transition metal doping [5] or photosensitization. Various p-block elements have been studied as dopants for the enhancement of photocatalytic properties, including nitrogen [6,7,8,9,10,11], carbon [12], sulphur [13,14], boron [15], fluorine [16], and combinations of several elements [17,18]. The exact mechanism of the photocatalytic activity enhancement is still under question, however it is generally accepted that band gap narrowing takes place due to mixing of the 2p states of the dopant with the 2p states of oxygen [19]. Alternatively, it has been suggested that the red shift observed as a result of p-element doping is due to the formation of colour centres, and that the doped materials may have completely different band gap electronic structures, as compared to undoped TiO_2_ [20]. Compared to N-doping, C-doping is a considerably less studied field, mainly due to fact that synthesis routes for C-doped TiO_2_ photocatalysts often involve multiple stages and/or unstable and costly reagents [19]. Nevertheless, a number of studies that are available indicate that carbon doping is a promising method for the enhancement of titania photocatalytic activity [21,22] due to band gap narrowing and the extended lifetime of photogenerated electrons and holes. The well-known fact that carbon-doped titania photocatalysts can have very different characteristics, with carbon found in interstitial, as well as in substitutional positions in the titania lattice, depending on the synthesis route, means that the choice of production technique is of particular importance in this case.

Titania thin films, as opposed to powdered or particulate photocatalysts, offer multiple advantages from the point of practical applicability, such as high recycle rates, ease of recovery, and separation from the media [23]. A number of deposition techniques are typically used for the deposition of titania and doped titania coatings, including chemical vapor deposition (CVD), sol gel, atomic layer deposition (ALD), hydrothermal synthesis, magnetron sputtering, etc. Of the methods given, magnetron sputtering is a process of particular industrial importance, as it is widely used for the production of high quality coatings for various commercial applications, including glazing products, thin film photovoltaics, data storage media, etc. [24,25]. It offers such advantages as excellent scalability, versatility, and high uniformity of the produced thin films. A number of techniques can be used for the deposition of C-doped TiO_2_ via magnetron sputtering, including using CO_2_ gas [26] or solid carbon targets as the source of carbon [27]. However, due to a limited number of studies available to date, it is still unclear how the choice of the dopant affects structural and photocatalytic properties of the C-doped titania coatings.

Therefore, the authors of the present work have performed a series of consecutive studies on the deposition of C-doped titania via reactive pulsed DC (pDC) magnetron sputtering, using different sources of carbon. This work describes the deposition of C-doped TiO_2_ using a mixture of CO_2_/O_2_ as the reactive gas at different ratios, followed by a study of the structural, morphological, and photocatalytic properties of these films. Carbon-doped coatings were compared to undoped titania coatings deposited under otherwise identical conditions.

## 2. Materials and Methods

### 2.1. Deposition of the Coatings

**Figure 1:**
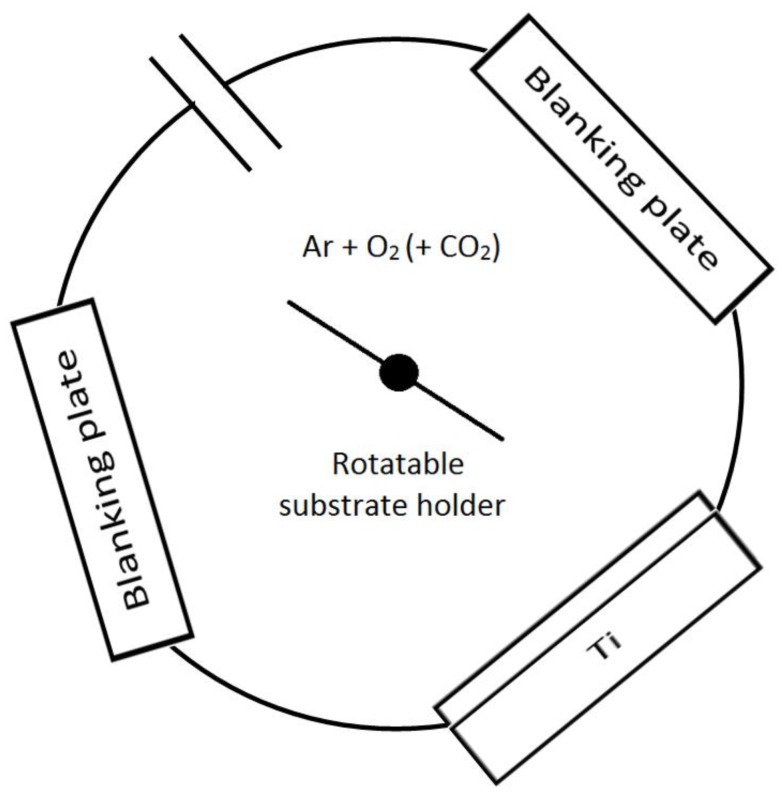
Schematic representation of the Teer UDP350 sputtering rig.

In this study the carbon-doped TiO_2_ films were prepared using a Teer Coatings UDP350 reactive pDC magnetron sputtering system (Teer Coatings Ltd., Droitwich, UK), without additional heating applied to the substrate. A schematic diagram of the rig is shown in Figure 1. In brief, the sputtering rig was fitted with a 300 mm × 100 mm directly cooled titanium metal target (99.5% purity) mounted onto a type II unbalanced planar magnetron, installed through the chamber wall. The titanium metal target was driven in pulsed DC mode using a dual channel power supply (Advanced Energy Pinnacle Plus, Fort Collins, CO, USA), at 100 kHz pulse frequency, and a duty cycle of 50%. The base pressure of the vacuum chamber before sputtering was 2.0 × 10^−5^ mbar or lower. All titanium dioxide films were deposited onto soda lime glass substrate materials. The substrates were ultrasonically pre-cleaned in propanol prior to deposition (all reagents used in this work were purchased from Sigma Aldrich, unless stated otherwise) and mounted on the rotatable substrate holder at 10 cm separation from the titanium target. During the sputtering process, the substrate holder was rotated at a speed of 10 rpm. The titanium metal target power was fixed at 1000 W for each run; the target was pre-sputtered in an argon plasma for 10 min prior to the deposition process to remove any oxide layer on the target surface (with the substrates shielded from the target), and the reactive sputtering time for each run was 2 h. The deposition runs were carried out in an Ar/O_2_ and Ar/O_2_/CO_2_ plasma for undoped and carbon-doped titanium dioxide films, respectively. The Ar flow was controlled by a mass flow controller and set at 15 sccm. The O_2_ flow was controlled by optical emission monitoring (OEM) at three different set points of 25%, 30%, and 35% of the full metal signal (FMS). To vary the carbon concentration, the CO_2_ flow rate was varied by the mass flow controller at different flows of 2.5, 5, and 7 sccm. The coatings were post-deposition isothermally annealed for 30 min at 873 K in air for crystal structure development and then allowed to cool gradually in air for 10–12 h to avoid the formation of thermal stresses in the coatings.

### 2.2. Characterization of the Coatings

The film thickness was measured via a Dektak™ stylus profilometer (Bruker, Billerica, MA, USA). The surface images of the coatings were obtained with scanning electron microscopy (SEM) (JEOL JSM-5410LV scanning microscope) (JEOL, Peabody, MA, USA). The micro structure and crystallinity of the thin films were investigated with a Panalytical Xpert powder X-ray diffractometer (XRD) (PANalytical Ltd., Cambridge, UK) operating with Cu Kα1 radiation at 0.154 nm in grazing incidence mode at a 3° angle of incidence, over a scan range from 20° to 70° 2θ and the accelerating voltage and applied current were 40 kV and 30 mA, respectively. The bulk composition of the thin films was characterized by energy dispersive X-ray spectroscopy (EDX, JEOL model JSM-5410LV scanning microscope) (JEOL, Peabody, MA, USA). The Ti 2p, O 1s, and C 1s core levels on the surface were measured by X-ray photoelectron spectroscopy (XPS) to examine the surface composition, electron binding energy, and bonding sites on the thin films. The XPS analysis was performed using an AMICUS photoelectron spectrometer (Kratos Analytical Ltd., Manchester, UK) equipped with an Mg Kα X-ray as the primary excitation source. The binding energy was referenced to the Ag 3d line at 368.2 eV for calibration. Curve fitting was performed using a Gaussian function with a Shirley background. The surface topography of the films was studied using atomic force microscopy (AFM) (Veeco, Plainview, NY, USA), specifically a Veeco NanoScope IV MultiMode AFM. The transmission data of the coatings were obtained using an Ocean Optics USB4000 UV-Visible spectrometer (Ocean Optics Inc., Oxford, UK), which was used in turn to evaluate the band gap energy according to the Tauc plot method, by plotting (*αhν*)^1/2^ as a function of *hν* and extrapolating the linear region to the abscissa (where *α* is the absorbance coefficient, *h* is Plank’s constant, *ν* is the frequency of vibration). The photoinduced wettability was evaluated at ambient temperature by analyzing the change in the water contact angles using a Theta Lite optical tensiometer (Biolin Scientific, Manchester, UK), while irradiating with UV light or visible light (identical to the light sources used for the methylene blue degradation described in the latter section). Prior to the experiments of contact angle measurements, the thin films were kept in the dark for 24 h.

### 2.3. Photocatalytic Activity Assessment

#### 2.3.1. Decomposition of Methylene Blue (MB)

The photocatalytic properties of the series of thin films deposited with different carbon concentrations were evaluated under UV and visible light illumination. For photocatalytic oxidation of methylene blue, samples of the same geometrical size (1.5 × 2.5 cm^2^) were immersed in 40 mL conditioning solution of methylene blue (concentration 1.5 μmol/L) in the dark at room temperature for 30 min to reach the adsorption-desorption equilibrium. The samples were then withdrawn from the conditioning solution and immersed into 40 mL of testing solution (concentration 1.5 μmol/L—pre-defined experimentally to detect the photocatalytic responses of all samples in a 1-hour experiment) with continuous magnetic stirring, while being irradiated with either UV or visible light for a total time of 1 h. During irradiation, the absorbance of methylene blue at 664 nm was monitored continuously with an Ocean Optics USB4000 spectrometer for one hour. Each coating was tested both under UV and visible light; 2 × 15 W 352 nm Sankyo Denki BLB lamps were used as the UV light source, while visible light was simulated by combining a fluorescent light source (2 × 15 W Ushio fluorescent lamps) with a Knight Optical 395 nm long pass UV filter. The emission spectra of the light sources used are given elsewhere [28,29]. According to the Lambert—Beer law, the concentration of the dye is proportional to the absorbance decay. The apparent first order rate constant, k_a_, was used as a quantitative characterization of the photocatalytic degradation rate of MB. Values for k_a_ were found from the gradient of the graph of A_t_/A_t=0_ versus experiment time (A_t=0_ and A_t_ are the peak absorbance values of the methylene blue solution at 664 nm at time 0 and time of experiment, respectively). A series of reference tests was performed prior to the photocatalytic activity measurements, which included tests with blank samples (soda-lime glass of the same geometrical size) under each light source, as well as tests of each sample in dark conditions, to prove that solution decolourization was caused by a photocatalytic reaction. As none of the reference tests showed more than 1% peak height decay in a 1 h experiment, their effect was neglected in further calculations.

#### 2.3.2. Decomposition of Stearic Acid (SA)

The stearic acid decomposition test was used for verification of the dye degradation results in this work. It is generally accepted that the stearic acid photocatalytic decomposition reaction pathway has no major intermediates, therefore it can be easily monitored through IR-observable species (SA disappearance or CO_2_ generation). A 0.1 M stearic acid solution in acetone was used for the test. The same sample size of the thin films, 1.5 cm × 2.5 cm, were spin-coated with 0.5 mL of stearic acid using an Osilla spin coater at 1000 rpm speed for 30 s. After spin coating the samples were kept in the dark for 1 h to ensure that the acetone had evaporated from the surfaces. As the stearic acid has three strong absorption peaks at 2958, 2923, and 2853 cm^-1^, the model pollutant decomposition was monitored here by Fourier transform infrared spectroscopy (FTIR), using a Perkin Elmer Spectrum Two IR spectrometer (Perkin Elmer, Waltham, MA, USA), in the range 2700−3000 cm^-1^ every 24 h. FTIR spectra were collected in absorbance mode and the amount of stearic acid on the surface of each sample was evaluated through the integrated area under the corresponding FTIR absorbance spectrum. Each sample was tested under both UV and visible light irradiation. The light sources used for irradiation of these samples were identical to the light sources used for the methylene blue decomposition tests.

## 3. Results and Discussion

### 3.1. Coatings Overview

An overview of deposition conditions, thickness information, and compositional results for the undoped and C-doped TiO_2_ coatings are summarized in Table 1. In terms of visual appearance, all coatings were uniform and optically transparent, with no visual signs of stresses. The color of the coatings varied from colorless (for undoped titanium dioxide samples) to pale yellow (for coatings deposited at low flow rates of CO_2_) and pale brown (for coatings deposited at high CO_2_ flow rates).

It was observed that the thickness of the carbon-doped films was considerably greater than that of the undoped titania; also, a gradual increase of coating thickness was observed with increasing carbon dioxide flow rate. The thickness difference between the corresponding coatings of the arrays deposited at 25% and 30% OEM setpoints was minimal, while array coatings at 35% OEM signal were considerably thicker, both in the case of undoped and C-doped samples. As established from earlier work, using a 25% OEM setpoint resulted in a titanium-to-oxygen ratio close to the stoichiometric 2:1. For undoped coatings deposited at higher OEM setpoints, the ratio of Ti:O was slightly higher. Carbon content in the doped coatings varied from 4 to 6.4 wt %. Unsurprisingly, coatings deposited at higher OEM setpoints (i.e., lower O_2_ content) were characterized with higher carbon content, as titanium preferentially reacts with oxygen, and creating oxygen deficiencies promotes carbon incorporation. Similar trends were seen as the carbon dioxide flow was increased.

**Figure 2:**
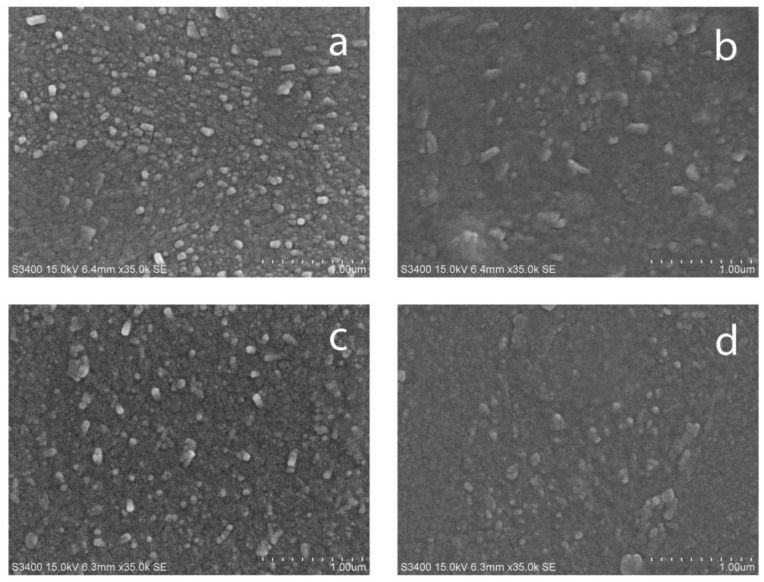
Scanning electron microscopy (SEM) images of selected titania and C-doped titania coatings deposited on glass substrate (**a**) T35; (**b**) T35C2.5; (**c**) T35C5; (**d**) T35C7.

Examples of SEM images for carbon-doped TiO_2_ and undoped TiO_2_ thin films deposited at 35% OEM setpoint are shown in Figure 2. It is evident that all films were characterized with relatively smooth surfaces consisting of small densely packed grains, as typically observed for titania and doped titania films deposited by reactive magnetron sputtering [24,30]; no obvious defects could be seen on the film surfaces.

### 3.2. Morphology of the Films (AFM)

**Figure 3:**
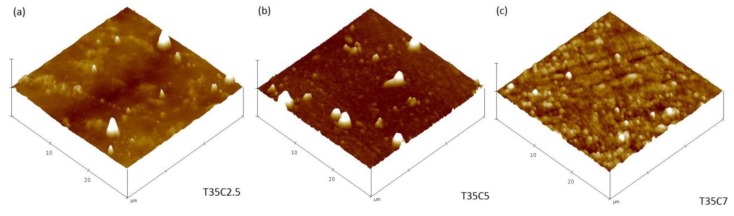
AFM images of selected C-doped coatings deposited on glass substrate (**a**) T35C2.5; (**b**) T35C5; (**c**) T35C7.

Morphological properties of the coatings (surface area and surface roughness) were studied with atomic force microscopy (AFM); the data on surface area and surface roughness values are presented in Table 2. The surface area of all deposited films was around 900 µm^2^; the variations of surface area between different samples was <1% for undoped and C-doped coatings. The roughness of the films obtained by AFM showed that coatings deposited at higher CO_2_ flow rates were characterized with higher roughness values. Examples of AFM images for carbon-doped TiO_2_ and undoped TiO_2_ thin films deposited at 35% OEM setpoint are shown in Figure 3. The differences in the surface topography of the films, from relatively smooth to the dense domains as a result of increasing carbon dioxide flow, are obvious from Figure 3. Despite the considerable difference in film thicknesses, similar values of surface area mean that the photocatalytic performance of the samples could be compared directly, rather than being normalized per unit of surface area, as is typically done when comparing the photocatalytic activity of samples with largely different surface areas [31].

### 3.3. Film Crystallinity (XRD)

**Figure 4:**
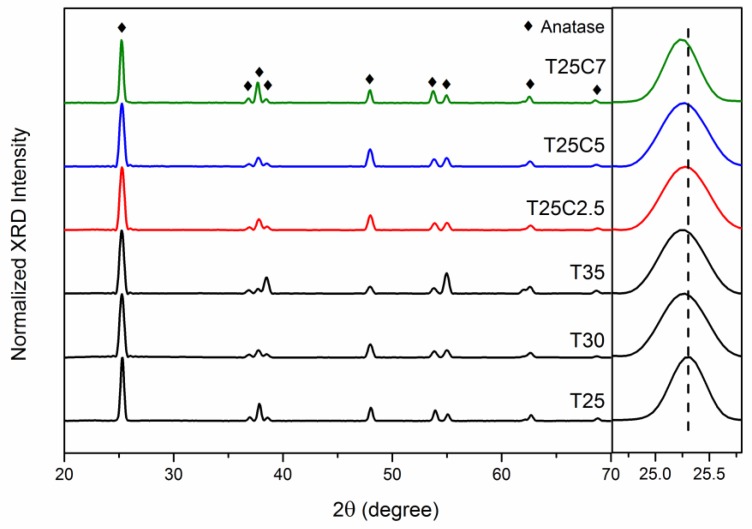
X-ray diffraction (XRD) patterns of selected C-doped and undoped TiO_2_ coatings (25% optical emission monitoring (OEM) setpoint) deposited on glass substrates and annealed at 873 K.

X-ray diffraction (XRD) analysis was employed to characterize the crystal structure of the undoped and C-doped thin films fabricated in this study. The XRD patterns of all samples before annealing were indicative of amorphous or weakly crystalline structures. After annealing, all samples were crystalline; sharp peaks observed at 2θ ca. 25.3°, 37.7°, 48.0°, and 55.0° on the diffraction patterns of all the analyzed samples can be identified as TiO_2_ anatase phase peaks (crystallographic card number 96-900-8215). Figure 4 shows the XRD patterns of undoped and carbon-doped TiO_2_ films deposited at 25% OEM setpoint after isothermal annealing in air at 873 K. No peaks that could be attributed to the other TiO_2_ crystal phases (rutile and brookite) were seen on the diffraction patterns. It can be seen that for the films produced at higher flows of carbon dioxide, the peaks were shifted to slightly lower values; this can be indicative of crystal lattice deformation as the result of carbon introduction. This shift is typically explained by the lattice distortion of TiO_2_ due to internal stress (carbon inclusion in this case), which caused the expansion of the unit cell volume [32]. Overall, the carbon incorporation in TiO_2_ does not cause any crystal phase transformations of TiO_2_, as all deposited films were in the anatase phase with a (101) preferred orientation, regardless of the OEM setpoint and carbon dioxide flow variations.

### 3.4. XPS Results

**Figure 5:**
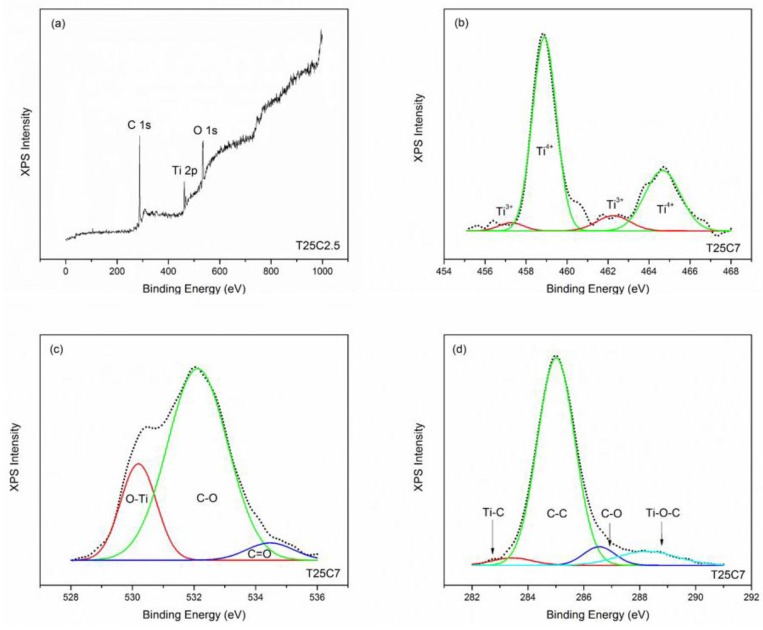
X-ray photoelectron spectroscopy (XPS) results of selected C-doped samples: (**a**) Survey spectrum (sample T25C2.5); (**b**) Ti 2p spectrum (sample T25C7); (**c**) O 1s spectrum (sample T25C7); (**d**) C 1s spectrum (sample T25C7).

To examine the species and the bonding states of all elements in the deposited undoped and carbon-doped TiO_2_ thin films, XPS spectra were recorded and deconvoluted. The XPS survey scans of all samples showed the presence of titanium and oxygen; a carbon peak was also seen for all carbon-doped TiO_2_ thin films (Figure 5a). Gaussian functions were used to deconvolute the Ti 2p, O 1s, and C 1s spectra. Ratios of titanium/oxygen on the surface calculated based on the XPS data were in good correlation with the bulk composition of the coatings estimated based on the EDX data (given in Table 1).

Figure 5b shows an example of a high resolution XPS spectra for Ti 2p (sample T25C7). The two strong peaks at binding energies of 458.8 and 456.4 eV were observed for all TiO_2_ thin films and can be assigned to Ti^4+^ 2p_3/2_ and Ti^4+^ 2p_1/2_, respectively [33]. Interestingly, for the films deposited at higher CO_2_ flows (5 and 7 sccm) the presence of Ti^3+^ can be seen on the XPS spectra—Ti^3+^ 2p_3/2_ and Ti^3+^ 2p_1/2_ at 457.3 and 462.3 eV, respectively. At the same time, no evidence of the presence of Ti^3+^ could be seen on the XPS spectra of the undoped and carbon-doped TiO_2_ thin films deposited at a low (2.5 sccm) flow of CO_2_. To quantify the observed effect, the ratio of Ti^3+^/Ti^4+^ was calculated for each sample (given in Table 2). Apparently, the ratio of the Ti^3+^ to Ti^4+^ states increased with increasing the CO_2_ flow rate. Ti^3+^ is typically reported as a surface defect of TiO_2_ that plays an important role for photocatalysis and the photoinduced hydrophilicity phenomenon. It can also act as an active site for oxygen desorption, prevent the electron-hole recombination process, and enhance visible light activity [34,35]. The Ti 3d peak at 455 eV that is typically assigned to Ti–C bonds could not be detected, but the existence of Ti-C bonds was confirmed on the high resolution C 1s spectrum [36]. Figure 5c shows an example of the high resolution O 1s spectrum (sample T25C7) and its deconvolution into three peaks. The peak at 530.2 eV is assigned to the lattice oxygen of TiO_2_ [37], while two other peaks at 532.1 and 534.6 eV correspond to C–O and C=O bonds, respectively [38]. For all carbon-doped TiO_2_ thin films, the binding energies exhibited a slight positive shift, which can be explained by carbon incorporation into the TiO_2_ lattice [39]. Figure 5d shows an example of the high resolution C 1s XPS profile of the carbon-doped TiO_2_ thin film (sample T25C7) deconvoluted into four peaks. The major broad peak at 285 eV is typically assigned to contaminant hydrocarbons (C–C) [40]. It is often reported that this peak disappears with increasing Ar^+^ etching time during the XPS measurement [41]. Contaminant carbon species are typically believed to not contribute to the visible light photocatalytic activity, however several works suggest that carbon may exist in the grain boundaries of TiO_2_ and therefore acts as a photosensitizer [42,43]. The peak at 283.4 eV corresponding to the titanium-carbon bond (Ti–C) [44] was observed for all carbon-doped TiO_2_ thin films. The presence of this peak indicates the substitution of oxygen atoms by carbon atoms as the result of carbon-doping (substitutional doping). The Ti–C bonding percentage was estimated using the relative areas of the peak at 283.4 eV and is shown in Table 2. This confirms the presence of carbon bonded in the TiO_2_ matrix. It can be concluded that the carbon atoms were substituted in some lattice oxygen sites not only at the surface but also in the bulk region, and the carbon concentration was increased with increasing CO_2_ flow rate. Two peaks at 286.6 eV and 288.3 eV can be attributed to the oxygen bound species, C–O and Ti–C–O, respectively, confirming the fact that some carbon was incorporated into the titania lattice in place of Ti atoms (interstitial doping) [22]. The atomic percentage of C doping in place of Ti atoms was estimated using the relative areas of the peaks at 288.3 eV and is given in Table 2. Reportedly, carbon substitution of both oxygen and titanium atoms, as well as the presence of the carbonate species (C–O) can contribute to enhanced visible light photocatalytic activity through band gap narrowing [19].

### 3.5. Optical Properties and Band Gap Calculations

**Figure 6:**
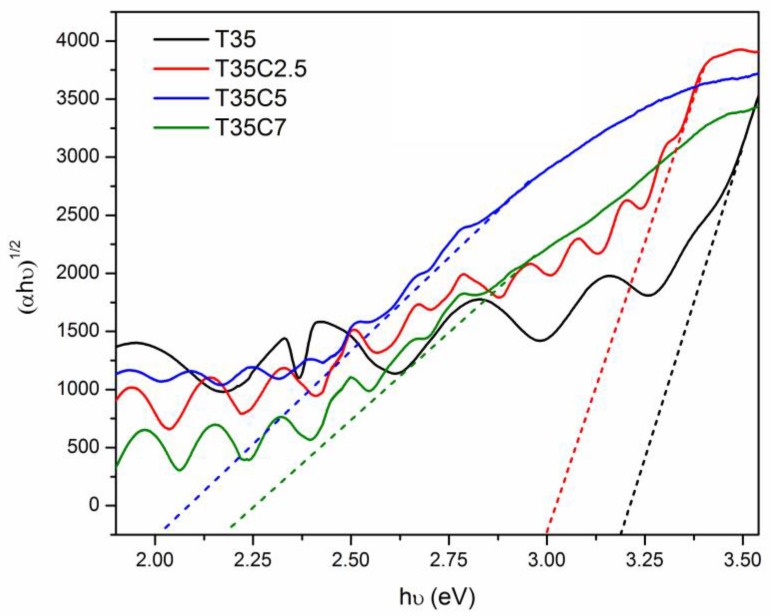
Examples of band gap calculations for selected C-doped and undoped titania samples (deposited at 35% OEM setpoint).

Optical band gaps of undoped and carbon-doped titania films were calculated using the Tauc plot method, as described in the Materials and Methods section. Graphical examples of the band gap calculation of selected samples are given in Figure 6 (films deposited at 35% OEM setpoint). Calculated band gap values of all the coatings studied are given in Table 2, along with the other analytical results. According to the calculation results, band gap values of undoped titania films deposited at 25%, 30%, and 35% OEM setpoint values were 3.22, 3.20, and 3.18 eV, respectively. These numbers are in good agreement with the data typically reported for anatase titania (ca. 3.2 eV). It is obvious from the data presented that increasing the CO_2_ flow rate, along with increasing the OEM setpoint resulted in remarkable red shifts of the absorbance edge. The large shift in band gap towards the visible light region (up to 1.2 eV—for sample T35C5, compared to undoped titania) is due to the inclusion of carbon into the TiO_2_ crystal lattice and subsequent band gap narrowing. Despite the different positions of the carbon atoms that may occur as a result of C-doping (incorporation in the TiO_2_ structure as Ti–C, Ti–C–O, and carbonate species), it is generally accepted that band gap narrowing is a result of the existence of mid-gap state(s) caused by mixing of the C 2p and O 2p states [19].

### 3.6. Wettability Measurements

Titanium dioxide-based surfaces are known for their ability to exhibit reversible wettability behaviour, becoming superhydrophilic upon sub-band gap photoexcitation and returning to their original wettability as the irradiation stops [45,46]. Water droplet contact angle (CA) measurements were performed here to study the wettability of the thin films upon UV and visible light irradiation; the results are given in Table 3. It can be seen that the undoped titania coatings and coatings produced at 2.5 sccm CO_2_ flow rate showed the highest values of CA both before and after irradiation. Thus, the contact angle values of sample T25 change from 92° to 18° and 55° after 60 min of UV and visible irradiation, respectively. While the initial contact angles of the undoped samples T30 and T35 were lower compared to T25 (53°), neither of these samples reached a superhydrophilic state after 1 h of irradiation. Whilst the initial values of the carbon-doped thin films produced at 2.5 sccm CO_2_ flow rate were comparable to those of undoped titania, the CA reduction under both UV and visible light irradiation was greater in this case. At the same time, wettability properties of the C-doped coatings produced at higher flow rates of CO_2_ (5 and 7 sccm) were remarkably different. These coatings exhibited superhydrophilicity (CA ca. 10°) prior to the light irradiation, with perfect wettability (a zero contact angle) after irradiation with either UV or visible light. However, this phenomenon was unsurprising, considering the presence of Ti^3+^ states observed with XPS for these samples. It is a frequently reported fact that an increased number of Ti^3+^ states on the surface results in considerably higher hydrophilicity [47]. Therefore, the lowest contact angle values both prior to and after irradiation in this study were observed for the samples that earlier showed the presence of Ti^3+^ states on their surfaces.

### 3.7. Photocatalytic Tests Results

**Figure 7:**
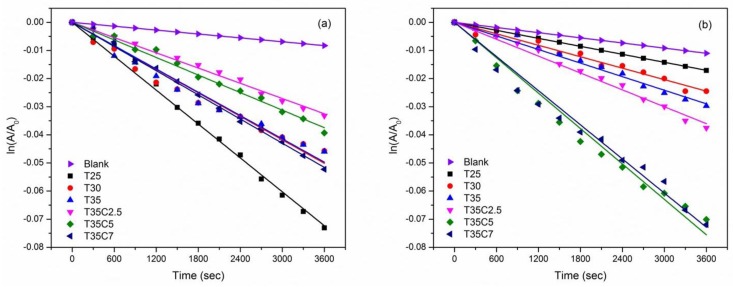
Kinetics plot of selected thin films of methylene blue degradation under (**a**) UV and (**b**) visible light irradiation.

To investigate the effect of carbon-doping on the photocatalytic activity of TiO_2_ thin films, two types of photocatalytic tests were used in this work; namely the decomposition of methylene blue and stearic acid. The MB decomposition reaction was approximated to pseudo first order kinetics and the reaction constants for the samples studied are given in Table 3. Examples of MB decay rates for the selected samples under UV and visible light irradiation are shown in Figure 7. The results under UV light irradiation are shown in Figure 7a. The methylene blue degradation rate constants for undoped TiO_2_ thin films were around 1.4–1.8 × 10 ^−5^ s^−1^. As can be seen from the data presented in Table 3, carbon doping did not help to improve UV light photocatalytic activity in this case; the rate constants of all C-doped samples were equal to or lower than those of the undoped titania samples. Figure 7b shows the selected methylene blue photocatalytic degradation under visible light irradiation. All undoped TiO_2_ thin films showed low rates of MB decay under visible light irradiation with kinetic constants below 1 × 10^−5^ s^−1^, and no significant variation of the kinetic constant values was seen for undoped titania coatings produced at different OEM setpoints. However, the C-doped samples showed higher photocatalytic activity under visible light irradiation. Generally, the ability to photodegrade MB under visible light irradiation increased with increasing OEM setpoint along with increasing CO_2_ flow rate. Of the samples studied, the highest MB decay rates under visible light irradiation were seen for samples T35C5 and T35C7. Given that the the band gap value is indicative of light absorbance, it is no surprise that samples T35C5 and T35C7 with the lowest band gap values (and consequently the highest visible light absorbance) proved the most active under visible light conditions.

**Figure 8:**
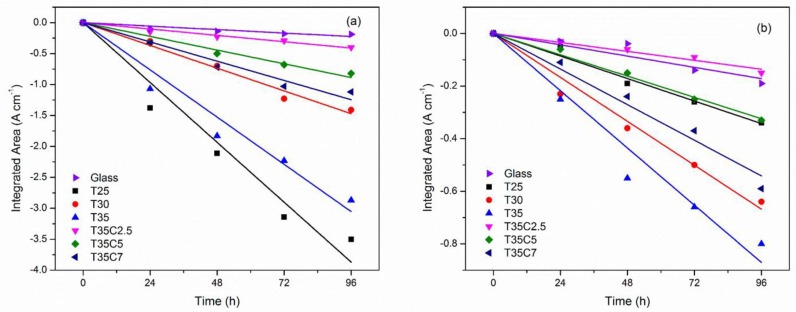
Plots of the integrated area changes of the FTIR spectra of the stearic acid peaks (3000–2700 cm^−1^) for selected C-doped and undoped TiO_2_ samples under: (**a**) UV light irradiation; (**b**) visible light irradiation.

Photocatalytic degradation of stearic acid, a common model pollutant for assessing self-cleaning properties of photocatalytic coatings, was monitored with FTIR through the disappearance of SA absorbance peaks. Reaction rate constants were calculated for the quantitative characterisation of the degradation process under UV and visible light (presented in Table 3). Prior to the experiments with photocatalytic surfaces, the test was carried out on pieces of uncoated glass. As no significant SA peak reduction was detected on plain glass under either of the two light sources used, it can be concluded that stearic acid films were stable under both UV and visible light. Examples of the decay of the integrated area under the SA absorbance spectrum for selected samples are given in Figure 8. From the data presented in Table 3, it is obvious that the highest degradation rates under UV light were recorded for undoped titania films, and in particular for T25. It is obvious that carbon doping had a detrimental effect on the coatings’ ability to degrade stearic acid. No improvement of photocatalytic activity for C-doped coatings could be seen under visible light either. While selected C-doped coatings outperformed sample T25, the overall rates of stearic acid degradation under visible light remained rather low.

Two types of photocatalytic tests were used in this work to cross-verify whether the incorporation of carbon into the titania lattice had a positive effect on photocatalytic activity. The stearic acid test was also used because the suitability of the dye degradation test is often questioned as a method for the determination of visible light activity [48]. Though no direct correlation between quantitative results of these two testing methods was found here, both sets of results were indicative of the fact that C-doping had a rather detrimental effect on the UV light photocatalytic activity of the samples, while improvement of the visible light activity was found only for two samples of the array (T35C5 and T35C7). As for visible light induced stearic acid degradation, the overall low levels of photocatalytic activity seen under visible light makes it difficult to quantify the activity precisely. Despite the fact that authors often report the correlation between the results of different photocatalytic tests [49,50], the examples of non-correlative results are also often reported [14]. This is evidence of the fact that some model pollutants can be decomposed much more easily than others.

Despite the widely published information that carbon doping is an efficient method of enhancement of UV and introduction of visible light photocatalytic activity, the present work confirms the fact that the properties of a photocatalytic material depend greatly on the production method, phase composition of TiO_2_, position of carbon in titania lattice, etc. It is frequently stressed in the literature that the exact mechanism of the enhancement of TiO_2_ photocatalytic properties via C-doping is not yet fully understood [19]. The majority of the work reporting significant improvements of photocatalytic activity for C-doped titania films report carbon found either in the interstitial [51,52] or in substitutional [26,53] position, while all of the C-doped films deposited in the present work reveal carbon in both positions in the same film. Similar results observed for nitrogen-doped samples may suggest that the presence of dopants in both positions may cancel out the effect, as compared to the samples where the dopant is found only in one of the positions [54]. It is also frequently mentioned in the literature that, for doped TiO_2_, excessive dopant may act as recombination sites, therefore lowering the photocatalytic activity. We assume that this hypothesis was the reason for the lower overall photocatalytic activity of the C-doped coatings produced here, as compared to undoped titania. This suggestion is supported by the fact that the UV-induced activity of C-doped photocatalysts for all samples was lower than that for undoped TiO_2_. Consequently, it may be concluded that the use of C-doped photocatalysts produced via magnetron sputtering with CO_2_ gas as the source of carbon is practical, only when the extension of the absorption spectrum into the visible range is more important than the efficient use of UV photons, and when the pollutants are relatively easy degradable [14].

## 4. Conclusions

The present work describes the deposition of C-doped titanium dioxide coatings onto glass substrates via reactive magnetron sputtering using mixtures of CO_2_ and O_2_ as the reactive gas. Undoped titania coatings were deposited under otherwise identical conditions for comparison purposes. It was demonstrated that variations of the OEM setpoint, as well as the CO_2_ flow rate, allowed variation of the carbon doping content in the coatings (ca. 4–6.5 wt %, according to the EDX results). Carbon films were found to be considerably thicker than the undoped titania films. Typically, for magnetron sputtered titania coatings, all as-deposited samples were amorphous; post-deposition annealing at 873 K resulted in anatase phase formation for all the films studied, as identified with XRD. XPS analysis revealed the existence of Ti^3+^ species for the coatings deposited at higher flows of CO_2_; carbon dopant atoms were found in both interstitial and substitutional positions in the titania lattice. Significant band gap narrowing was achieved as the result of C-doping, with BG values as low as 2.02 eV, compared to ca. 3.2 eV for undoped titania. In contrast to undoped titania films, C-doped samples showed higher photoinduced wettability. Carbon-rich coatings exhibited superhydrophilicity prior to irradiation; the latter phenomenon was found to correlate with the presence of Ti^3+^ species. Photocatalytic activity was tested using dye (MB) and stearic acid decomposition tests; UV-induced photocatalytic activity of the undoped TiO_2_ films was superior to C-doped ones for both pollutants. Visible light photoactivity of carbon-rich samples was found to be improved in the case of MB degradation, while stearic acid decomposition results under visible light were low for all the films studied. Overall, it can be concluded that the above method is practical for producing coatings when the extension of the absorption spectrum into the visible range is more important than the efficient use of UV photons and the target pollutants are easily degradable.

## Figures and Tables

**Table 1:** Summary of the deposition conditions and compositional properties of undoped and C-doped titanium dioxide films on glass substrate.

Sample ID	Optical Emission Monitoring (OEM) Setpoint, % FMS	CO_2_ Flow, sccm	Thickness, nm ^a^	Ti, at. % ^b^	O, at. % ^b^	C, at. % ^b^
T25	-	-	490	34.9	65.1	-
T25C2.5	25	2.5	820	32.9	62.5	4.5
T25C5	25	5	860	32.9	61.6	5.0
T25C7	25	7	870	35.2	59.4	5.5
T30	30	-	510	40.9	59.1	-
T30C2.5	30	2.5	820	33.9	62.0	4.1
T30C5	30	5	870	33.5	60.8	5.3
T30C7	30	7	910	35.8	58.6	5.6
T35	35	-	600	40.2	59.8	-
T35C2.5	35	2.5	840	33.6	60.7	4.7
T35C5	35	5	1300	34.1	59.8	6.1
T35C7	35	7	1500	36.7	56.9	6.4

^a^ Stylus profilometry results; ^b^ energy dispersive X-ray spectroscopy (EDX) results.

**Table 2:** Summary of the deposition conditions and surface properties of undoped and C-doped titanium dioxide films on glass substrate.

Sample ID	Surface Roughness, nm ^a^	Surface Area, µm^2 a^	Ti^3+^/Ti^4+ b^	Ti–C, at. % ^b^	C–O, at. % ^b^	Ti–C–O, at. % ^b^	Band Gap, eV ^c^
T25	5.7	902	-	-	-	-	3.22
T25C2.5	6.0	901	-	0.06	0.28	0.28	3.13
T25C5	4.2	901	0.02	0.09	0.72	0.39	3.15
T25C7	13.0	902	0.03	0.18	0.34	0.48	3.08
T30	6.0	901	-	-	-	-	3.20
T30C2.5	5.8	901	-	0.08	0.39	0.16	3.04
T30C5	5.8	901	0.02	0.13	0.55	0.42	3.05
T30C7	10.0	902	0.04	0.27	0.49	0.34	2.63
T35	6.2	911	-	-	-	-	3.18
T35C2.5	12.8	902	-	0.11	0.37	0.51	3.00
T35C5	11.0	902	0.02	0.18	0.74	0.32	2.02
T35C7	12.4	902	0.06	0.31	0.49	0.46	2.17

^a^ Atomic force microscopy (AFM) results; ^b^ Calculation based on the X-ray photoelectron spectroscopy (XPS) results; ^c^ Calculation based on transmittance data.

**Table 3:** Results of wettability measurements and photocatalytic tests of C-doped and undoped Titanium dioxide (TiO_2_) coatings annealed at 873 K.

Sample ID	Initial CA, deg.	CA after 1 h UV, deg.	CA after 1 h vis, deg.	Kinetic Constant, k			
				MB UV, k × 10^5^, s^−1 a^	MB vis, k × 10^5^, s^−1 a^	SA UV, k × 10^2^, A cm^−1^ h^−1 b^	SA vis, k × 10^2^, A cm^−1^ h^−1 b^
T25	92	18	55	1.8	0.5	4.0	0.4
T25C2.5	60	10	15	1.8	1.5	0.8	0.6
T25C5	11	~0	~0	1.3	1.0	1.1	0.3
T25C7	11	~0	~0	1.4	0.8	1.0	0.2
T30	53	13	20	1.4	0.9	1.6	0.7
T30C2.5	58	13	16	0.8	0.9	1.2	0.1
T30C5	11	~0	~0	0.7	1.5	1.3	0.2
T30C7	10	~0	~0	0.8	1.4	0.7	0.2
T35	54	10	22	1.4	0.9	2.3	0.9
T35C2.5	63	12	14	0.9	1.0	0.4	0.1
T35C5	10	~0	~0	1.0	2.1	0.9	0.3
T35C7	10	~0	~0	1.4	2.0	1.3	0.6

^a^ MB UV/Vis—Methylene blue degradation under UV or visible light, respectively; ^b^ SA UV/Vis—Stearic acid degradation under UV or visible light, respectively.
